# Incidences of colorectal adenomas and cancers under colonoscopy surveillance suggest an accelerated “Big Bang” pathway to CRC in three of the four Lynch syndromes

**DOI:** 10.1186/s13053-024-00279-3

**Published:** 2024-05-13

**Authors:** Pål Møller, Saskia Haupt, Aysel Ahadova, Matthias Kloor, Julian R. Sampson, Lone Sunde, Toni Seppälä, John Burn, Inge Bernstein, Gabriel Capella, D. Gareth Evans, Annika Lindblom, Ingrid Winship, Finlay Macrae, Lior Katz, Ido Laish, Elez Vainer, Kevin Monahan, Elizabeth Half, Karoline Horisberger, Leandro Apolinário da Silva, Vincent Heuveline, Christina Therkildsen, Charlotte Lautrup, Louise L Klarskov, Giulia Martina Cavestro, Gabriela Möslein, Eivind Hovig, Mev Dominguez-Valentin

**Affiliations:** 1 Department of Tumour Biology, Institute of Cancer Research, The Norwegian Radium Hospital, Oslo, 0379 Norway; 2 Engineering Mathematics and Computing Lab (EMCL), Interdisciplinary Center for Scientific Computing (IWR), Heidelberg University, Heidelberg, Germany; 3 Data Mining and Uncertainty Quantification (DMQ), Heidelberg Institute for Theoretical Studies (HITS), Heidelberg, Germany; 4 Department of Applied Tumour Biology, Institute of Pathology, Heidelberg University Hospital, Heidelberg, Germany; 5 Clinical Cooperation Unit Applied Tumour Biology, German Cancer Research Centre (DKFZ), Heidelberg, Germany; 6 Institute of Medical Genetics, Division of Cancer and Genetics, Cardiff University School of Medicine, Heath Park, Cardiff, CF14 4XN UK; 7 Department of Clinical Genetics, Aalborg University Hospital, Aalborg, 9000 Denmark; 8 Department of Biomedicine, Aarhus University, Aarhus, DK-8000 Denmark; 9 Clinical Cancer Research Center, Aalborg University Hospital, Aalborg, Denmark; 10 Faculty of Medicine and Health Technology, Tays Cancer Center, Tampere University, Tampere University Hospital, Tampere, Finland; 11 Department of Gastrointestinal Surgery, Helsinki University Central Hospital, University of Helsinki, Helsinki, Finland; 12 Applied Tumour Genomics, Research Program Unit, University of Helsinki, Helsinki, Finland; 13 Faculty of Medical Sciences, Newcastle University, Newcastle upon Tyne, NE1 7RU UK; 14 Dept. of Quality and Coherence, Aalborg University Hospital, Aalborg, 9000 Denmark; 15 Department of Clinical Medicine, Aalborg University Hospital, Aalborg University, Aalborg, 9100 Denmark; 16 Hereditary Cancer Program, Institut Català d’Oncologia-IDIBELL, L; Hospitalet de Llobregat, Barcelona, 08908 Spain; 17 Manchester Centre for Genomic Medicine, Division of Evolution, Infection and Genomic Sciences, University of Manchester, Manchester University NHS Foundation Trust, Manchester, M13 9WL UK; 18 Department of Molecular Medicine and Surgery, Karolinska Institutet, Stockholm, 171 76 Sweden; 19 Dept Clinical Genetics, Karolinska University Hospital, Solna, Sweden; 20 Genomic Medicine, The Royal Melbourne Hospital, Melbourne, Australia; 21 Department of Medicine, University of Melbourne, Melbourne, Australia; 22 Department of Gastroenterology, Medical Center, Faculty of Medicine, Hebrew University of Jerusalem, Hadassah, Israel; 23 Gastroenerolgy institute, Sheba medical center and Faculty of medicine Tel Aviv university, Tel Aviv, Israel; 24 Lynch Syndrome & Family Cancer Clinic, Centre for Familial Intestinal Caner, St Mark’s Hospital, London, UK; 25 Gastrointestinal Cancer Prevention Unit, Gastroenterology Department, Rambam Health Care Campus, Haifa, Israel; 26 Department of Surgery, Universitätsmedizin Mainz, Mainz, Germany; 27 Hospital Universitário Oswaldo Cruz, Universidade de Pernambuco, Recife, Brazil & SEQUIPE, Recife, Brazil; 28 Gastro Unit, The Danish HNPCC Register, Copenhagen University Hospital – Amager and Hvidovre, Copenhagen, Denmark; 29 Dept of Pathology, Copenhagen University Hospital - Herlev and Gentofte, Herlev, Denmark; 30 Dept of Clinical Medicine, University of Copenhagen, Copenhagen, Denmark; 31 Gastroenterology and Gastrointestinal Endoscopy Unit, Division of Experimental Oncology, IRCCS San Raffaele Scientific Institute, Vita-Salute San Raffaele University, 20132 Milan, Italy; 32 Surgical Center for Hereditary Tumors, University Düsseldorf, Ev. Bethesda Khs, Duisburg, Germany; 33 Centre for bioinformatics, Department of Informatics, University of Oslo, Oslo, Norway; 34 Department of Clinical Genetics, Aarhus University Hospital, DK 8000 Aarhus, Denmark

**Keywords:** MSI, *MLH1*, *MSH2*, *MSH6*, *PMS2*, dMMR, Lynch syndromes, Colorectal, cancer, Adenoma, Colonoscopy, Sojourn time

## Abstract

**Background:**

Colorectal cancers (CRCs) in the Lynch syndromes have been assumed to emerge through an accelerated adenoma-carcinoma pathway. In this model adenomas with deficient mismatch repair have an increased probability of acquiring additional cancer driver mutation(s) resulting in more rapid progression to malignancy. If this model was accurate, the success of colonoscopy in preventing CRC would be a function of the intervals between colonoscopies and mean sojourn time of detectable adenomas. Contrary to expectations, colonoscopy did not decrease incidence of CRC in the Lynch syndromes and shorter colonoscopy intervals have not been effective in reducing CRC incidence. The prospective Lynch Syndrome Database (PLSD) was designed to examine these issues in carriers of pathogenic variants of the mis-match repair (*path_MMR*) genes.

**Materials and methods:**

We examined the CRC and colorectal adenoma incidences in 3,574 *path_MLH1, path_MSH2, path_MSH6* and *path_PMS2* carriers subjected to regular colonoscopy with polypectomy, and considered the results based on sojourn times and stochastic probability paradigms.

**Results:**

Most of the *path_MMR* carriers in each genetic group had no adenomas. There was no association between incidences of CRC and the presence of adenomas. There was no CRC observed in *path_PMS2* carriers.

**Conclusions:**

Colonoscopy prevented CRC in *path_PMS2* carriers but not in the others. Our findings are consistent with colonoscopy surveillance blocking the adenoma-carcinoma pathway by removing identified adenomas which might otherwise become CRCs. However, in the other carriers most CRCs likely arised from dMMR cells in the crypts that have an increased mutation rate with increased stochastic chaotic probabilities for mutations. Therefore, this mechanism, that may be associated with no or only a short sojourn time of MSI tumours as adenomas, could explain the findings in our previous and current reports.

## Background

There are four dominantly inherited microsatellite instability (MSI) Lynch syndromes, caused by pathogenic variants of the four *MMR (path_MMR)* genes *MLH1, MSH2, MSH6 and PMS2* (OMIM **#**120,435; **#**609,310; #614,350 and #614,337) [1, 2]. Deletions of the *EPCAM* tail #613,244 epigenetically silence the *MSH2* gene and in this report are grouped together with OMIM **#** 120,435. The four *path_MMR* variant groups have different penetrance (incidence of any cancer) and expressivities (incidences of cancer in the different affected organs), causing different overall risks of death related to any cancer and to cancer in specific organs [1]. In the normal population, it is postulated that most CRCs arise through biallelic somatic *APC* mutations initiating an adenoma that may develop into a carcinoma as a result of acquiring additional driver mutations, and less often through an initiating mutation in *CTNNB1* [3, 4].

Assuming that CRCs in *path_MMR* carriers develop from adenomas, and observing that carriers do not have many adenomas, the paradigm of an accelerated adenoma-carcinoma pathway was established in the Lynch syndromes [5]. The mechanism for the accelerated pathway was assumed to be mediated via deficiency of MMR proteins (dMMR) in MSI adenomas [6].

It soon became clear that colonoscopy with polypectomy (below referred to as colonoscopy) every third year did not prevent CRC in carriers as had been hoped and more frequent colonoscopies were advocated [7]. However, large studies reported that shorter intervals between colonoscopies reduced neither CRC incidences (in *path_MMR* carriers) nor its stage at diagnosis [8, 9]. To assess the incidence and prognosis of CRC in *path_MMR* carriers subjected to colonoscopy as is advocated in clinical guidance, the European Hereditary Tumour Group (EHTG, www.ehtg.org, at that time named The Mallorca Group) initiated the Prospective Lynch Syndrome Database (PLSD, www.plsd.eu ). When comparing CRC incidences in carriers followed up in PLSD who were subjected to colonoscopy surveillance with other cohorts who did not receive colonoscopy surveillance, the CRC incidences in *path_MLH1, path_MSH2* and *path_MSH6* carriers were either increased or not reduced in PLSD, depending on which retrospective segregation analyses were used [10–13]. By contrast, CRC incidences in *path_PMS2* carriers followed up in PLSD were reduced [14]. A recent statement by the EHTG discusses that these findings support emerging knowledge that adult *path_MLH1* and *path_MSH2* carriers have huge numbers of MMR-deficient crypts (dMMR) arising in their colons [15–17]. It appears that these may be controlled or destroyed by the immune system or may develop into CRC without an intermediate adenoma stage. Further, it is now also clear that not only dMMR crypts, but also dMMR/MSI CRCs may be removed by the host immune system, as demonstrated by the success of immune checkpoint blockade therapy [1, 18, 19].

Previous studies using the PLSD have provided new data and insights into the associations between colonoscopy intervals, incidences of CRC by age and times from a normal surveillance colonoscopy to diagnosis of CRC, its stage at diagnosis and prognosis. In this report we add new data on the incidences of colorectal adenomas and their relationship to the incidences of CRC.

### Aims

The aims were to determine the incidences of colorectal adenomas by age and gene in *path_MMR* carriers, compare these with the incidences of CRC and evaluate the results in relation to previously reported paradigms [3, 5] and our previously suggested hypotheses regarding pathways to CRC in the Lynch syndromes [1, 6, 20] thereby better informing future clinical guidelines for prevention and treatment.

## Materials and methods

The PLSD was initiated in 2012 and the first report on CRC incidences, stratified by *MMR* gene was published in 2015 [21]. Since then, the number of records in the PLSD has tripled, while the incidences of CRC by age, gene and gender have remained similar, leading us to the conclusion that the incidences initially reported have been validated in the newly recruited cases. The methods used and our most recent results have been described in detail [1, 22–24] (www.plsd.eu). In the current study, as detailed in our previous reports, cumulative incidences of CRC were calculated from 25 to 75 years of age as time to first CRC in carriers who had not had CRC prior to or at their first prospectively planned and completed colonoscopy. Overall 10 year survival following CRC was estimated.

Polyps were grouped as adenomas or non-adenomatous polyps, and adenomas were grouped as either advanced or not (advanced adenomas = those > 10 mm diameter and/or villous morphology and/or high-grade dysplasia). The total number of adenomas removed up to and including at the last colonoscopy was noted for each *path_MMR* carrier. We compared incidences of adenomas by age and gene using standard linear regression analysis. We also report on the presence or absence of adenomas at the last colonoscopy undertaken before CRC was diagnosed and the time between that last colonoscopy and the diagnosis of CRC. Carriers were grouped into those who had ever had adenoma(s) and those who had not and cumulative incidences of CRC were compared, stratified by age and by gene.

We discuss the results using the concept of mean sojourn time which makes no assumptions regarding mechanisms for what is observed [25], as is usual when examining the effectiveness of screening in cancer prevention. This may be explained as follows: ‘*The parameter estimated in practice in screening programs is the average sojourn time over all disease cases, usually referred to as the mean sojourn time. A long mean sojourn time indicates a good potential for screening. The shorter the sojourn time, the more frequently screening has to take place in order to be effective. If the mean sojourn time is very short, screening may not be worthwhile at all*.’ [26]. Screening colonoscopy to prevent CRC is based on the adenoma-carcinoma paradigm [5] with detection and removal of tumours at the adenoma stage. Here we consider sojourn time as the time for which the tumour is detectable as an adenoma by colonoscopy. PLSD records discrete observations in time that are assumed to be Poisson-distributed [27] and that may be considered based on the paradigm of stochastic causative processes [28, 29].

## Results

The required details were available for 3,574 carriers who were followed for total of 38,735 years after their initial (prevalence) colonoscopies. The mean ages of male and female carriers in each *path_MMR* group were similar at the end of the study (range 52.8 to 57.0 years, Table 1). The 2,444 (68%) carriers never had a colorectal adenoma detected at colonoscopy and 1,130 (32%) had one or more colorectal adenomas detected, all of which were removed.

**Table 1:** Number of carriers by sex and gene, average age and number of adenomas with 95% CI, number of adenomas by grade, and total number of adenomas

Gene	Sex	Number	Sum follow-up years (mean for carriers)	Mean age (95% CI)	Number of adenomas		
					Total	Mean (95% CI)	Advanced
***Path_MSH2***	M	574	6,206 (10.8)	53.0 (1.2)	554	0.97 (0.16)	66
	F	661	6,689 (10.1)	53.6 (1.2)	519	0.79 (0.15)	59
***Path_MLH1***	M	649	8,880 (13.7)	54.4 (1.1)	519	0.80 (0.13)	50
	F	719	10,126 (14.1)	56.0 (1.1)	544	0.76 (0.15)	52
***Path_MSH6***	M	346	2,680 (7.7)	57.0 (1.7)	311	0.90 (0.21)	33
	F	426	3,123 (7.3)	56.2 (1.6)	243	0.57 (0.15)	12
***Path_PMS2***	M	85	415 (4.9)	56.0 (3.4)	39	0.46 (0.22)	0
	F	114	616 (5.4)	52.8 (2.7)	38	0.33 (0.18)	4
**Sum**		**3,574**	**38,735**		**2,767**		**276**

**Fig. 1:**
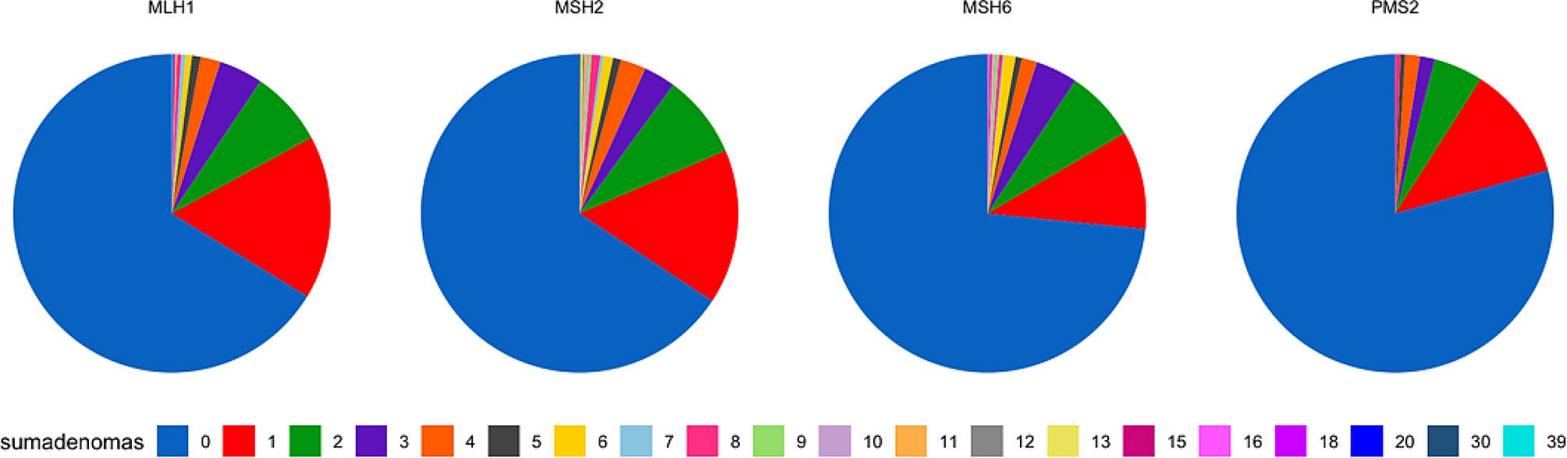
Pie-charts showing total number of colorectal adenomas by genetic variant

**Fig. 2:**
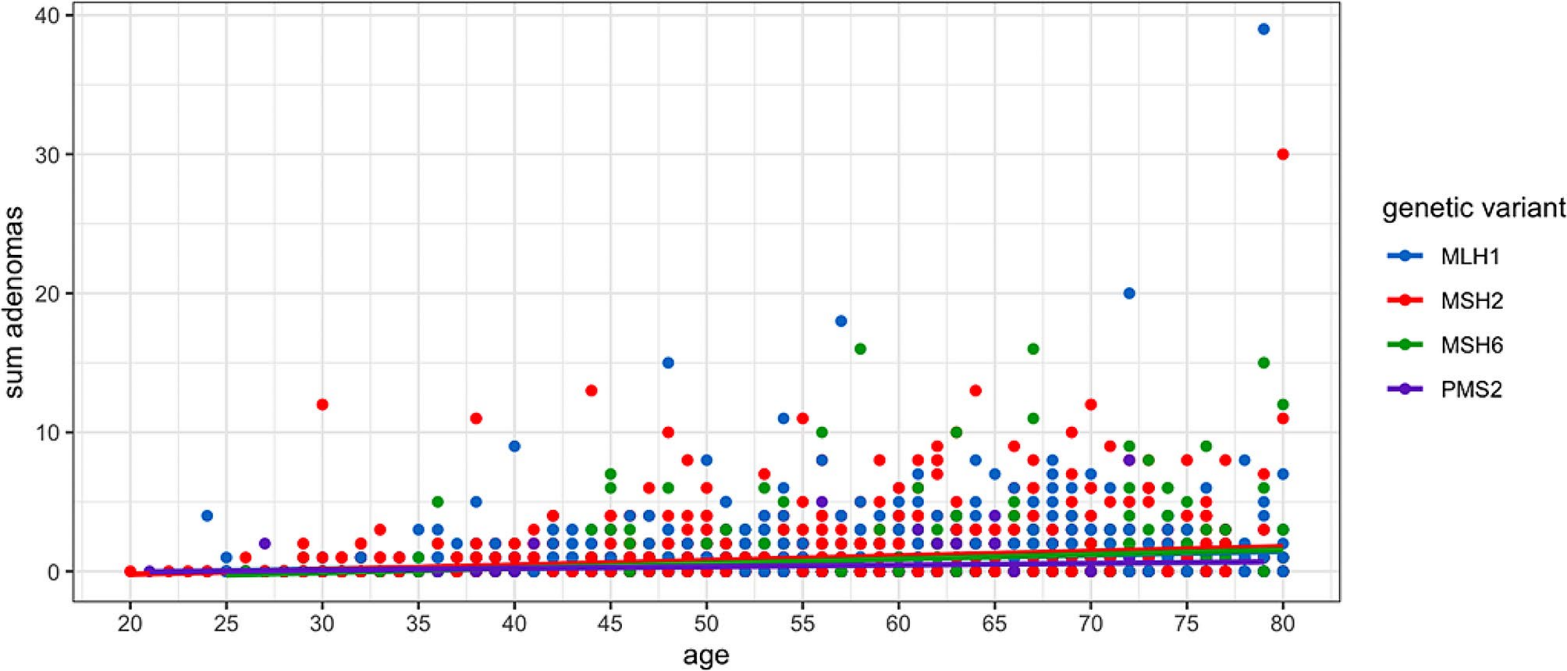
Standard linear regression on the total number of colorectal adenomas by genetic variant and age for age up to 80 years at last examination. *Path_PMS2* carriers had less than the others (*p* < 0.01). Only a few at any age and with any genetic variant had multiple colorectal adenomas. Regression line for MLH1 is hidden under MSH6.

The detailed findings, grouping carriers by gene, their total number of adenomas (advanced and not advanced) and the numbers of non-adenomatous polyps (categorised as unclassified, hyperplastic, serrated, juvenile or hamartomatous), total number of polyps and average age in each group are given in Table 2; Fig. 1. The standard linear regression analysis of the number of adenomas by carrier and by gene is presented in Fig. 2.

**Table 2:** Number of carriers by gene and sum adenomas, also indicating numbers of adenomas, unclassified polyps, hyperplastic, serrated, juvenile, hamartomas, total number of polyps and average age in group

Gene	N	Number adenomas in each carrier in group (row)	Number of carriers within group (row) with other polyps, by polyp type					
			**unclassified**	**hyperplastic**	**serrated**	**juvenile**	**hamartomas**	Mean age
*Path_MLH1*	907	0	0	0	0	0	0	53.6
	227	1	0	2	0	0	0	54.8
	105	2	0	0	0	0	0	60.8
	61	3	0	0	0	0	0	63.3
	28	4	0	2	0	0	0	59.8
	12	5	1	0	0	0	0	61.3
	9	6	1	0	0	0	0	67.4
	6	7	1	0	0	0	0	71.5
	6	8	4	0	0	0	0	66.7
	1	9	0	0	0	0	0	40.0
	1	11	0	0	0	0	0	54.0
	2	15	2	0	0	0	0	64.5
	1	18	0	0	2	0	0	57.0
	1	20	0	0	2	0	0	72.0
	1	39	0	0	12	0	0	79.0
*Path_MSH2*	812	0	0	0	0	0	0	50.8
	194	1	0	0	0	0	0	55.7
	105	2	0	0	0	0	0	57.1
	40	3	0	0	0	0	0	63.3
	32	4	0	5	0	0	0	59.3
	10	5	0	0	0	0	0	68.2
	12	6	0	0	0	0	0	65.7
	4	7	0	0	0	0	0	65.8
	11	8	0	6	2	0	0	64.8
	3	9	0	0	1	0	0	66.3
	3	10	0	2	0	0	0	60.0
	3	11	0	3	0	0	0	57.7
	2	12	0	4	9	0	0	50.0
	3	13	0	0	0	0	0	63.7
	1	30	0	4	0	0	0	80.0
*Path_MSH6*	567	0	0	0	0	0	0	53.6
	77	1	0	0	0	0	0	62.7
	56	2	0	2	0	0	0	65.0
	33	3	0	0	0	0	0	64.7
	12	4	0	4	0	0	0	72.1
	5	5	0	0	1	0	0	62.4
	9	6	0	6	0	0	0	64.3
	1	7	0	0	0	0	0	45.0
	3	8	0	4	0	0	0	82.7
	2	9	0	2	1	0	0	74.0
	2	10	2	0	0	0	0	59.5
	1	11	0	0	0	0	0	67.0
	1	12	0	1	0	0	0	80.0
	1	15	0	0	0	0	0	79.0
	2	16	0	4	0	0	0	62.5
*Path_ PMS2*	158	0	0	0	0	0	0	52.7
	23	1	0	2	1	0	0	57.5
	10	2	0	0	0	0	0	62.3
	3	3	0	0	0	0	0	58.0
	3	4	0	0	0	0	0	69.0
	1	5	0	4	0	0	0	56.0
	1	8	0	0	0	0	0	72.0

**Fig. 3:**
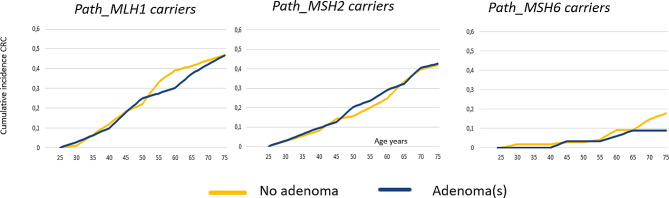
Cumulative incidences of CRC in *path_MLH1, path MSH2* and *path_MSH6* carriers with and without colorectal adenoma(s)

Annual incidences of both CRC and colorectal adenomas increased with age (Table 2; Fig. 3) as previously reported by others [30].

There was no statistically significant difference in the prevalence of adenomas in *path_MLH1* versus *path_MSH2* carriers but both *path_MSH6* and *path_ PMS2* carriers were significantly less likely to have any adenomas than were *path_MLH1* or *path_MSH2* carriers (*p* < 0.001). In contrast, among carriers with adenomas *path_MLH1*, *path_MSH2* and *path_MSH6* all carriers had similar regression curves for numbers of adenomas, while *path_PMS2* carriers less frequently had multiple adenomas (Figs. 2 and 3, *p* < 0.01). Advanced adenomas were with one exception seen only in carriers with multiple adenomas. Serrated adenomas were rarely seen (Table 2). If, as we propose, adenomas may disappear spontaneously in *path_MMR* carriers because of targeting by the host immune system, the cumulative incidences of adenomas shown in Fig. 3 may be underestimates.

As seen in Tables 2 and 34%, 34%, 27% and 21% of *path_MLH1*, *path_MSH2, path_MSH6* and *path_PMS2* carriers, respectively had one or more adenomas and only a minority (5%, 7%, 5% and 4%, respectively) had more than 3 adenomas. There were therefore insufficient numbers to calculate meaningful cumulative incidences of CRC by age in carriers with multiple adenomas. Among the 3,574 carriers included in the study, 2,293 (64%) had no CRC before or at the first planned and carried out (prevalence round) colonoscopy. They were followed-up with regular colonoscopy for total of 26,213 years and 239 CRCs were diagnosed prospectively in carriers from 25 to 75 years of age. These included 151, 85, 12 and 1 CRCs in carriers of *path_MLH1, path_MSH2, path_MSH6* and *path_PMS2*, respectively (Table 3).

**Table 3:** Numbers, follow-up years and prospectively detected CRCs in carriers with and without adenomas in last colonoscopy before CRC; and without CRC before or at first colonoscopy (prevalence round), by genetic variants

	Gene	Number carriers	Sum follow-up years	Number CRC
Carriers with adenoma(s)	*Path_MLH1*	554	7,567	82
	*Path_MSH2*	518	5,125	47
	*Path_MSH6*	402	2,775	10
	*Path_PMS2*	116	538	0
Sum		1,590	16,005	
Carriers without adenoma	*Path_MLH1*	329	5,778	69
	*Path_MSH2*	231	3,068	28
	*Path_MSH6*	114	1,134	3
	*Path_PMS2*	29	228	0
Sum		703	10,208	
Total		2,293	26,213	239

Table 4 shows the numbers of carriers with and without adenomas at the last colonoscopy before diagnosis of CRC, together with the number of months elapsed between the last colonoscopy and diagnosis of CRC. In each group of *path_MMR* carriers, the majority had no adenomas at the last colonoscopy and the months elapsed were similar in carriers with and without adenomas.

**Table 4:** Number having no polyps, adenomas or other polyps (not classified or otherwise classified) detected at last colonoscopy before CRC diagnosed by gene, age at CRC diagnosed, and months since last colonoscopy before CRC was diagnosed

Gene	adenoma	*N*	Mean months before (95% CI)	Mean age (95% CI)
*Path_MLH1*	No	83	26.7 (2.8)	53.1 (2.8)
	Yes	35	29.4 (8.5)	52.7 (3.7)
*Path_MSH2*	No	59	35.1 (8.9)	52.6 (3.0)
	Yes	25	27.9 (9.7)	54.0 (4.5)
*Path_MSH6*	No	8	20.6 (7.7)	58.8 (7.5)
	Yes	5	28.6 (14.6)	52.0 (15.1)

As seen in Fig. 3, there were no differences in the cumulative incidences of CRC by gene in *path_MLH1, path_MSH2* and *path_MSH6* carriers stratified by the presence or absence of adenomas (the curves are overlapping and no confidence estimates or calculation of p-values were needed to reach this conclusion).

No CRC was diagnosed prospectively in *path_PMS2* carriers below 75 years of age.

There was no significant difference in 10-year overall survival after first prospectively detected CRC in carriers with and without adenomas (88% and 81%, *p* > 0.05 respectively).

## Discussion

The findings of this study were as follows:

- the majority of *path_MMR* carriers did not develop any colorectal adenoma(s) during follow up;
- colonoscopy appeared to prevent CRC only in *path_PMS2* carriers; and.
- there was no association between the incidences of adenomas and CRCs in any group of carriers.
- In contrast to previous reports on carriers not subjected to colonoscopy [14], no CRC was diagnosed prospectively in *path_PMS2* carriers below 75 years of age, to the combined conclusion that colonoscopy as undertaken had prevented CRC in *path_PMS2* carriers in the current study.
- The lower incidences of colorectal adenomas in *path_PMS2* carriers compared to *path_MLH1, path_MSH2* and *path_MSH6* carriers, may suggest that some adenomas in the latter groups are attributable to the *path_MMR* genotypes.

Considered by the sojourn time paradigm, the PLSD findings are in keeping with a theory that colonoscopy blocks the adenoma-carcinoma pathway and that the CRCs observed had no or short sojourn times as adenomas. CRCs arising by the *CTNNB1* pathway appear to have no sojourn time as adenomas [3], are not frequent in *path_MMR* carriers [31], and may be specific for *path_MLH1* carriers [32–34].

Our findings pose the following question: if the adenoma-carcinoma pathway that is initiated by *APC* mutation [3, 6, 31] is blocked by colonoscopy and the carcinogenetic pathway has to include an early mutation causing an increased cell proliferation rate [3, 20], how have the observed CRCs emerged? There are many published pathophysiological, biochemical and DNA analyses of adenomas and CRCs. Based on these, we have previously suggested an additional pathway in *path_MMR* carriers in which dMMR crypts may trigger the adenoma-carcinoma pathway [6]. A recent mathematical analysis of the most frequently mutated genes in CRCs supported this theory [20].

Descriptions of tumours reflect different time-points in different carcinogenetic processes and, when tumours are removed for observation, subsequent events that might have occurred are blocked (“*the very act of measurement or observation directly alters the phenomenon under investigation”)* [35]. There is no way of fully characterising an adenoma or CRC without altering it. As no direct observations of the order of events in carcinogenetic processes are possible, the suggested linear models that are based on biological findings in adenomas and CRCs are assumed, not observed. In addition, mutations observed in adenomas and CRCs are influenced by selection (in *path_MMR* carriers including survival in the face of the host immune system’s continuous efforts to identify and remove the tumours) and genetic drift which may involve interacting stochastic processes.

When re-evaluating our previous proposal of three linear pathways to CRC in *path_MMR* carriers [6] and adding the findings reported in this paper, we conclude that the following model is probable: The main pathway to CRC in *path_MMR* carriers may be triggered by a second-hit in the wild-type MMR allele leading to a dMMR crypt and increasing the acquisition of mutations in driver genes [3, 20] in a continuous chaotic stochastic process which may lead to CRC. The probability of any dMMR clone leading to CRC seems very low. Adult *path_MLH1* and *path_MSH2* carriers may have thousands of dMMR crypts during life [36] but develop no or very few dMMR adenomas or MSI cancers. Information on dMMR crypts and dMMR adenomas incidences in *path_MSH6* and *path_PMS2* carriers is limited. While our previous mathematic modelling was consistent with the linear theory that dMMR crypts initiate the adenoma-carcinoma pathway, it did not indicate that there necessarily is an adenoma stage before an MSI CRC [20].

**Fig. 4:**
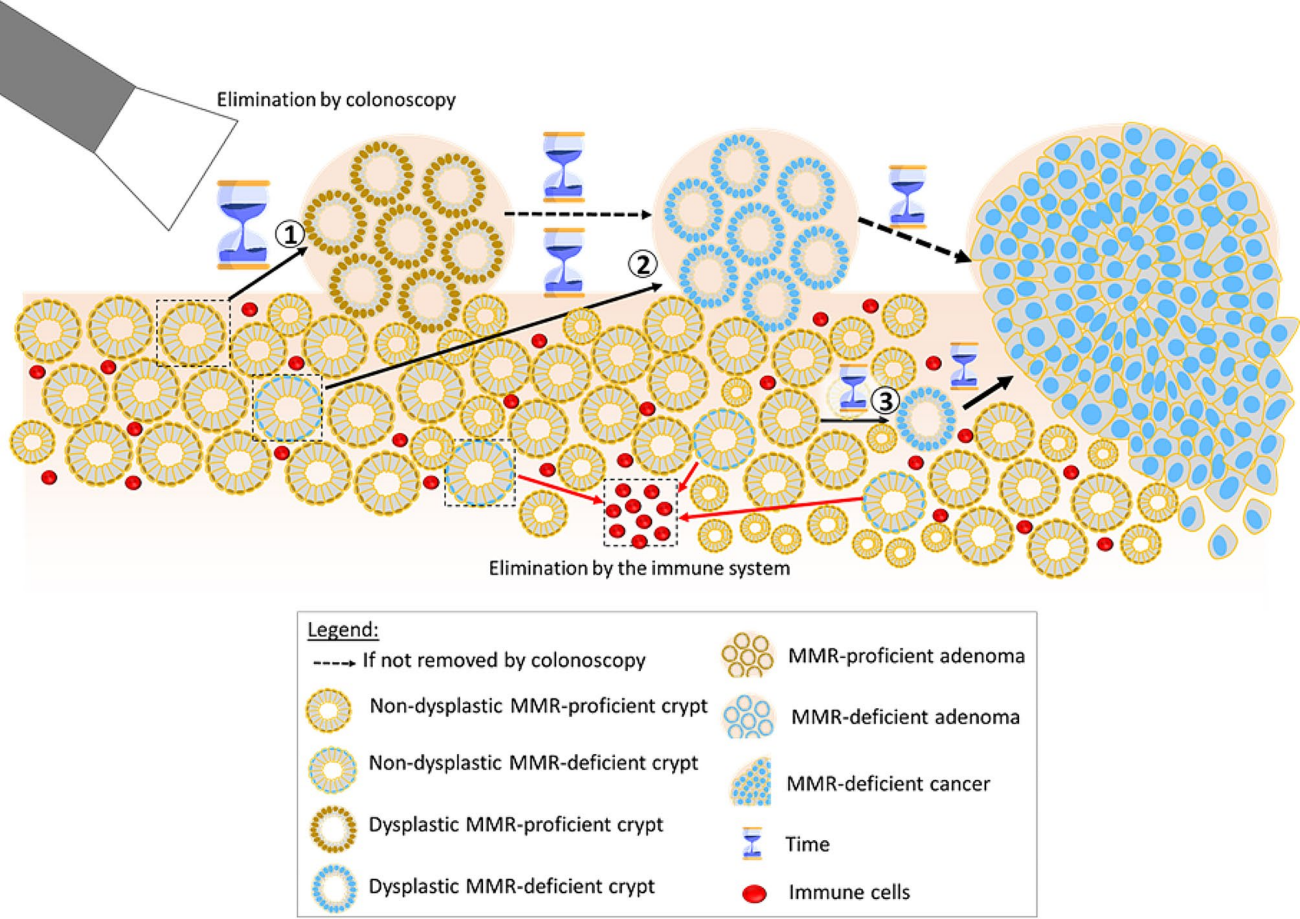
Possible pathways to CRC in LS modified from [6]. Carcinogenesis in LS follows a stochastic process where two major selection pressures apply: selection through conferred growth advantage and counter-selection through the immune system. MMR-deficient crypts may undergo immune elimination (arrows at the bottom central part of the graphic pointing to an empty space left by the eliminated crypt and now filled with immune cells), and MMR-deficient adenomas and cancers might be eliminated by the immune system, too (not shown by arrows). However, cancer may still arise via at least three pathways depicted here. Cancers arising through Pathway 1 may have a longer sojourn time as adenomas, as they spend part of their progression as MMR-proficient adenomas and only later acquire MMR deficiency. At this stage, Pathway 1-cancers can be prevented by colonoscopy. If not prevented, further mutations could accumulate eventually leading to cancer. Cancers arising through Pathway 2 may have shorter sojourn time as adenomas compared to Pathway 1, as they develop from initially MMR-deficient crypts acquiring further mutations to develop into MMR-deficient adenomas, thus not spending time as MMR-proficient lesions. However, the sojourn time as adenoma in Pathway 2 is longer compared to cancers arising through Pathway 3. The latter cancers likely have no sojourn time as adenomas and arise upon a single somatic hit (cnLOH) activating beta-catenin and inactivating MLH1. According to the current knowledge, this pathway is therefore specific for MLH1 carriers. Red arrows indicate regression due to counterselection by the immune system, black arrows indicate progression due to gained growth advantage, dotted black arrows indicate hypothetical progression if removal by colonoscopy did not take place Attribution: Hourglass element is an image by freepik

**Fig. 5:**
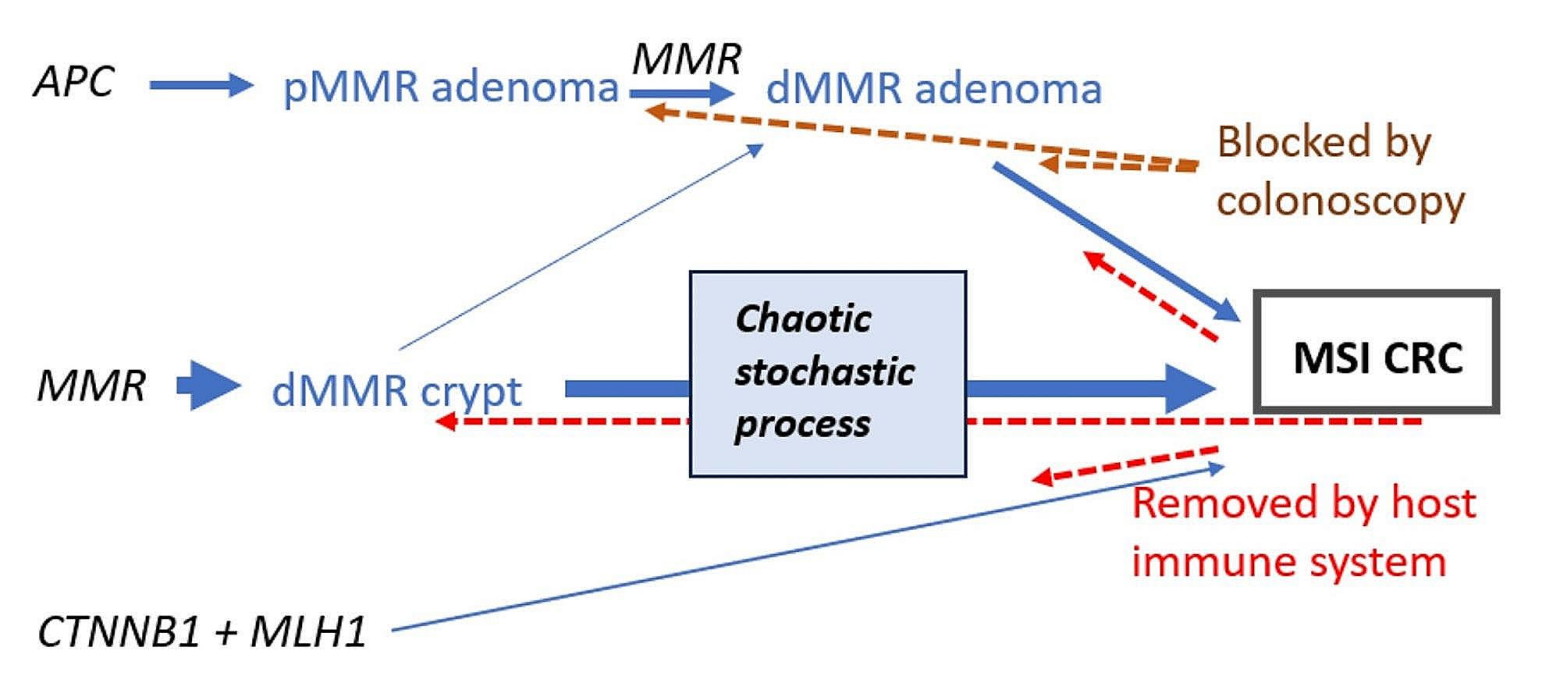
Simplified diagram of possible pathways to MSI CRC in *path_MMR* carriers, modified from [6, 20], compliant with Fig. 4 and specifying the accelerated chaotic stochastic process Big Bang theory [37] as discussed in the text *APC* inactivation occurs prior to loss of the wild-type MMR allele resulting in adenoma initiation. Subsequent loss of the second MMR allele in the adenoma generates a dMMR clone with increased risk of progression to cancer The initial event is loss of the second MMR allele resulting in a dMMR crypt causing an accelerated stochastic chaotic probability of mutations (that is greater for *path_MLH1* and *path_MSH2* compared to *path_MSH6* and only marginally raised in *Path_PMS2*). Many different driver genes may be mutated and in different orders [38], causing no or only a short sojourn time of tumours as adenomas. A dMMR cell may also become a dMMR adenoma In *path_MLH1* carriers, a single LOH event at chromosome 3p22 may inactivate the wild-type alleles of the co-located *CTNNB1* and *MLH1* genes (in a cell that has already sustained a single *CTNNB1* mutation, usually at codon 41 or 45 in Exon3). This specific initiating event may occur in up to 40% of CRC in *Path_MLH1* carriers [32] Regular colonoscopy may block the adenoma-carcinoma pathway. dMMR/MSI crypts, adenomas and cancer might be removed by the host immune system. Mutated genes in black. pMMR adenoma: MMR proficient adenoma. dMMR adenoma: MMR deficient adenoma

The findings of the current study indicate that CRCs developing from dMMR crypts may have no or only short sojourn times as adenomas (Fig. 4). This is in keeping with a chaotic stochastic pathway initiated by the dMMR/MSI crypts and is similar to ‘the Big Bang’ theory suggested for CRC in the general population [37]. Instead of an accelerated adenoma-carcinoma pathway, our current results may indicate an accelerated stochastic pathway accounting for most CRCs in *path_MSH2, path_MLH1* and *path_MSH6* carriers, but not *path_PMS2* carriers (Fig. 5). A similar theory with mutations causing invasive growth and spread before clonal expansion has been suggested causing cancer in *path_BRCA1/2* carriers leading to the same consequences of multiple subclones in one tumour, some of which having no or short sojourn time in adenoma stage [38]. Both these two theories (the one suggesting a Big Bang, the other considering different mutations in different parts of the tumours at different times), are based on recognizing high probabilities for stochastic mutations and may cause the same observed consequences. A theoretical discussion on complex probabilities as causative factors for events that are yet to happen is outside the scope of this report. For simplicity, we have used the well-known and easy-to-remember annotation ‘Big Bang’ in our title to indicate a causative chaotic stochastic probability where the outcome may not be predicted and for which the outcome that is observed does not elucidate exactly the events that led to that outcome.The theory we propose may explain our previously reported findings of similar or increased incidences of CRC in *path_MMR* carriers receiving regular colonoscopy compared to historical retrospective cohorts and the lack of any association between time since last colonoscopy and stage of CRC at diagnosis [9, 10, 39] as well as the current finding of no association between incidences of adenomas and CRCs. Noting that more than one mutation may occasionally occur simultaneously in a chaotic stochastic process [37], and that the host immune system may remove invasive cancer cells (as demonstrated by the success of immunotherapy for MSI CRCs in *path_MMR* carriers), all of our previously reported findings that were in conflict with the accelerated adenoma-carcinoma hypothesis may now be explained. Our current findings and theory are consistent with colonoscopy blocking the adenoma-carcinoma pathway but do not support the accelerated adenoma-carcinoma paradigm as the major cause of increased CRC incidence in *path_MMR* carriers.

Colonoscopy is subject to time-trends. Our results are based on surveillance including colonoscopy that was carried out over several decades in the collaborating centres and they may be considered to represent largely historical observations. More recent and sophisticated colonoscopy techniques may detect smaller adenomas and/or adenomas with different morphological patterns. Staining/chromoendscopy might visualise dMMR crypts and guided or unguided machine learning (artificial intelligence) may provide new ways of interpreting digitalized colonoscopy images [40, 41]. The effects of these advances may be a longer mean sojourn time which could increase the probability of colonoscopy preventing CRC. One study using frequent colonoscopies with advanced techniques reported a high incidence of small adenomas and a low incidence of CRC, indicating that improved colonoscopy may reduce CRC incidences [42]. None of this is in conflict with our suggested theory of stochastic processes being the main driver for the CRCs we observed.

There are numerous reports on adenomas in the Lynch syndromes but few reports describing the relationships between adenomas and CRCs in carriers subjected to colonoscopy and none that report cumulative incidences of CRC or include sufficient *path_MSH6* and *path_PMS2* carriers to arrive at meaningful conclusions. Our findings were similar to those in a study of 112 carriers from Cleveland, Ohio among whom CRC was diagnosed only in *path_MLH1* and *path_MSH2* carriers [43]. A further recent report from Toronto on 429 carriers found that more than half of CRCs in *path_MMR* carriers occurred in patients without adenomas [44]. The methods used did not allow direct comparison with our results. The authors concluded that fewer CRCs were diagnosed in carriers with adenomas when intervals between colonoscopies were shorter, but they did not report findings for short intervals between colonoscopies for the majority of carriers who did not have adenomas. Furthermore, they scored advanced adenomas as CRC and censored observation time when advanced adenomas were found. They did not report cumulative incidences of CRC. The reduced CRC incidence in the minor fraction of carriers who had multiple adenomas and who received more frequent colonoscopy is not in conflict with the minor fraction of carriers with multiple adenomas in our study (Table 2; Figs. 1 and 2) who were too few in number to have an impact on the averages we report or to calculate separate cumulative CRC incidences by age.

A study on 136 carriers from Houston, reported on associations between the presence of adenomas, their stages and CRC [45]. As in the Toronto study, advanced adenomas and CRCs were grouped together and CRC cumulative incidences in carriers with and without adenomas were not reported. Corresponding with our results, they observed no difference in the likelihood of advanced adenomas or CRCs for any of the measured covariates.

In North-America there has been a discussion on prophylactic colectomy in *path_MMR* carriers [46]. But none of the above reports that grouped advanced adenomas and CRC together indicated whether they did so because colectomy was undertaken when advanced adenomas were found, and none is in conflict with our suggested theory of stochastic processes being the main driver for the CRCs we observed.

The strengths of our study include the large number of carriers and follow-up years available and that no assumption on the mechanisms underlying CRC was included in the ascertainment criteria. Determination of the results of stochastic processes in time requires sufficient numbers of observations in all strata of interest and sufficient observation time for all strata to reach endpoints. The PLSD was designed to investigate why the observed effects of colonoscopy could not be explained by the accelerated adenoma-carcinoma paradigm alone. Its aim of considering empirical observations in a sufficiently large number of carriers who were subjected to colonoscopy in relation to theories regarding carcinogenetic pathways has been achieved in the current study. CRC incidences in all groups of carriers at all ages have been similar from the first to the most recent PLSD reports, while the numbers of carriers and observation years had tripled, indicating that the CRC incidences in carriers receiving colonoscopy surveillance that are reported by the PLSD are valid. We find no reason to doubt the cumulative incidences of CRCs in carriers with and without adenomas in the current report.

The main weakness of our study is that, to our knowledge, it is the only study so far to compare cumulative incidences of adenomas with cumulative incidences of CRCs in all age groups of carriers, thus our results have not been validated in an independent replication cohort. We therefore plan to continue this study and to collect data on adenomas from those PLSD contributors who have so far only reported cancers to PLSD. All the information required is likely recorded in medical history of subjected patients but retrieving and contributing these data will require time, ethical approval and funding, resources that may not be available in all contributing centres.

The aim of this report was to gain knowledge of adenoma and CRC incidences in path_MMR carriers subjected to regular colonoscopy. We hope the findings may contribute to the refinement of clinical guidelines for the prevention of CRC in *path_MMR* carriers. Our previous empirical observations and the theories we have suggested to interpret them have been used as arguments for current clinical guidelines [47, 48], but in light of our recent and current findings, some current recommendations may need to be reconsidered.

1st, since colonoscopy prevents CRC in young *path_PMS2* carriers the advice to postpone colonoscopy in young adult *path_PMS2* carriers until 35 years of age should be questioned.

2nd, since undertaking colonoscopy more frequently than every three years may not reduce CRC incidences in *path_MLH1* and *path_MSH2* carriers, the rationale for more frequent colonoscopies should be questioned.

3rd, the very minor fraction of *path_MMR* carriers who have multiple adenomas will be identified at colonoscopy and may be followed more intensively. The driving forces causing their multiple adenomas remain unclear, and empirical knowledge on effects of interventions in this group are limited.

## Conclusions

The PLSD was designed to address why colonoscopy with polypectomy did not reduce CRC incidence as had been assumed by accelerated adenoma-carcinoma model for CRC in *path_MMR* carriers. Based on combined and concomitant epidemiological and biological findings we propose an alternative model in which there is an accelerated chaotic stochastic pathway to MSI CRC from the dMMR crypt which may cause invasive tumours with no or short sojourn time in adenoma stage. We note, with interest and excitement, that in addition to its applicability in CRC, our model may be relevant to the much wider spectrum of cancers that characterise the four Lynch syndromes.

## Data Availability

The tables are the raw data grouped as indicated. Figure 1 is based on Table 2. The complete SQL code for how the grouped data underlying Fig. 3 was extracted from the MySQL database is published in reference #22. Anonymized data underlying Fig. 2, the annual incidences by age group underlying Fig. 3 are available upon request, both pending written agreement that none of these will be used for any other purpose than confirming the figures to be correct.
